# Thoracoscopy-Guided vs. Ultrasound-Guided Paravertebral Block in Thoracoscopic Surgery: A Non-Inferiority Randomized Trial

**DOI:** 10.3390/jcm14238493

**Published:** 2025-11-30

**Authors:** Seok Beom Hong, Kwanyong Hyun, Hoon Choi

**Affiliations:** 1 Department of Thoracic and Cardiovascular Surgery, Seoul St. Mary’s Hospital, College of Medicine, The Catholic University of Korea, Seoul 06591, Republic of Korea; seok_beom@naver.com; 2 Department of Thoracic and Cardiovascular Surgery, St. Vincent’s Hospital, College of Medicine, The Catholic University of Korea, Seoul 06591, Republic of Korea; 3 Department of Anesthesiology and Pain Medicine, Seoul St. Mary’s Hospital, College of Medicine, The Catholic University of Korea, Seoul 06591, Republic of Korea; hoonie83@catholic.ac.kr

**Keywords:** thoracic surgery analgesia, paravertebral block, thoracoscopy-guided analgesia, ultrasound-guided analgesia, video-assisted thoracoscopic surgery

## Abstract

**Background:** Thoracic paravertebral block (TPVB) is an established component of multimodal analgesia and enhanced recovery pathways following thoracoscopic lung resection. A surgeon-performed, thoracoscopy-guided approach has been proposed to improve intraoperative workflow, but high-quality comparative data are limited. **Methods:** In this single-center, randomized, non-inferiority trial, adult patients undergoing thoracoscopic lobectomy or segmentectomy received either thoracoscopy-guided TPBV (T-TPVB) conducted by surgeons or ultrasound-guided TPBV (U-TPVB) conducted by anesthesiologists. Blocks were performed at the end of surgery at the T4 and T7 vertebra levels, using 10 mL of 0.5% ropivacaine per level. The primary outcome was dynamic pain during coughing at 1–6 h postoperatively (visual analog scale, VAS). Secondary outcomes included resting/dynamic pain scores, opioid consumption over 48 h, block-related complications, and procedural time. **Results:** Seventy-three patients were included in the intention-to-treat analysis. Mean dynamic VAS scores at 1–6 h were 3.3 (T-TPVB) and 3.1 (U-TPVB), with a mean difference of 0.2 (95% CI: −0.3 to 0.7), meeting the non-inferiority criterion (margin 0.9). Secondary outcomes, including pain trajectories and opioid consumption, were comparable between groups. Procedural time was significantly shorter in the T-TPVB group, with no differences in complication rates. **Conclusions:** Surgeon-performed thoracoscopy-guided TPVB was non-inferior to the standard ultrasound-guided technique for early postoperative pain after thoracoscopic lung resection. Both methods provided comparable analgesic efficacy and safety profiles, while T-TPVB significantly reduced procedural time. This approach may support streamlined perioperative workflows and optimize enhanced recovery protocols in thoracic surgery. (Trial registration number, KCT0006471).

## 1. Introduction

Video-assisted thoracoscopic surgery (VATS) is now the standard approach for many thoracic procedures, offering less surgical trauma, shorter hospital stays, and faster recovery than open thoracotomy [1]. However, moderate to severe postoperative pain remains common, especially within the first 24 h, due to intercostal nerve injury, chest tubes, and soft tissue trauma [2]. This can impair respiratory function, limit effective coughing, and increase the risk of atelectasis and pneumonia [3]. Optimizing perioperative analgesia is thus central to Enhanced Recovery After Surgery (ERAS) protocols in thoracic surgery [1].

Thoracic paravertebral block (TPVB) provides analgesia comparable to epidural but with fewer side effects, such as hypotension and urinary retention [4,5]. Compared to intercostal or erector spinae plane blocks (ESPB), TPVB has generally demonstrated more consistent early analgesia and lower opioid consumption in earlier trials [6,7], although recent evidence from the OPtriAL randomized trial reported that intercostal nerve block provided non-inferior pain control compared with thoracic epidural analgesia [8]. It may also reduce the risk of chronic post-thoracotomy pain [9]. As a result, PROSPECT and ERAS guidelines recommend ultrasound-guided TPVB (U-TPVB) for minimally invasive thoracic surgery [1,10]. However, U-TPVB is operator-dependent, requires expertise, and may be challenging in patients with obesity, scoliosis, or limited ultrasound windows [4]. These barriers can limit implementation in some resource-constrained settings [3,5].

Thoracoscopy-guided TPVB (T-TPVB) is an emerging alternative that allows direct visualization and injection into the paravertebral space under thoracoscopic vision at the end of surgery [11,12,13]. Potential advantages include real-time confirmation of local anesthetic spread, shorter procedural time, and seamless integration into the surgical workflow [14,15]. While preliminary reports suggest feasibility and efficacy, high-quality comparative data are limited [14,15].

We hypothesized that T-TPVB would be non-inferior to U-TPVB for early postoperative analgesia after VATS lobectomy or segmentectomy. We therefore conducted a randomized, non-inferiority trial comparing surgeon-performed T-TPVB and anesthesiologist-performed U-TPVB, with dynamic pain during coughing at 1–6 h postoperatively as the primary outcome, reflecting early functional pain.

## 2. Methods

### 2.1. Study Design

This single-center, randomized, non-inferiority trial at a tertiary academic center compared the analgesic efficacy of surgeon-performed T-TPVB and anesthesiologist-performed U-TPVB in patients undergoing VATS lobectomy or segmentectomy. Approved by the Institutional Review Board (IRB No. KC21EISI0204; 9 July 2021), all participants provided written informed consent. The trial was registered with the Clinical Research Information Service (CRIS; registration No. KCT0006471; 20 August 2021), a WHO primary registry. Enrollment was from 29 September 2021 to 25 December 2022. The trial adhered to the Declaration of Helsinki and was reported in accordance with the CONSORT 2010 guidelines, including non-inferiority extensions.

### 2.2. Participants

Eligible patients were aged 20–75 years, scheduled for elective VATS lobectomy or segmentectomy, with ASA physical status I–III. Exclusion criteria included refusal to participate, emergency surgery, chronic analgesic use, defined as regular daily or near-daily use of opioid or non-opioid analgesics for more than three months prior to surgery, communication difficulties, psychiatric illness, allergy to local anesthetics, coagulopathy, and conversion to thoracotomy.

### 2.3. Randomization and Blinding

Patients were randomized 1:1 to receive T-TPVB or U-TPVB using a computer-generated sequence with variable block sizes (4 and 6) stratified by an independent researcher. Allocation concealment used sequentially numbered, opaque envelopes. Blinding of proceduralists was not feasible; however, patients and the outcome assessor were blinded. Block success (sensory blockade and dermatomal spread) was evaluated in the post-anesthesia care unit (PACU) by the procedural team after emergence from anesthesia. All postoperative pain and complication assessments were subsequently performed by a separate blinded observer who was not involved in block placement or sensory testing, ensuring maintenance of blinding.

### 2.4. Interventions

Both blocks were performed at the T4 and T7 levels with 10 mL of 0.5% ropivacaine per level to ensure adequate dermatomal coverage across surgical and chest tube sites, consistent with previous studies reporting more reliable segmental spread with multilevel injection [16].

In the T-TPVB group, blocks were performed at the end of surgery using a utility port under thoracoscopic guidance. The needle was advanced approximately 0.5 cm into the paravertebral space, immediately lateral to the vertebral body and deep to the parietal pleura [14,15]. After confirming negative aspiration, 10 mL of 0.5% ropivacaine HCl (Naropin injection; Mitsubishi Tanabe Pharma Korea Co., Ltd., Seoul, Republic of Korea) was injected at both the T4 and T7 levels. Pleural elevation was used to confirm the solution spread (online Supplemental Video S1).

In the U-TPVB group, the blocks were similarly performed at the end of the surgery. The paravertebral space was located laterally to the spinous process using real-time ultrasonography. After negative aspiration, 10 mL of 0.5% ropivacaine was injected at T4 and T7, and pleural displacement confirmed the solution spread (online Supplemental Video S2). This technique followed established protocols validating U-TPVB as a non-inferiority comparator [4,17,18].

Both the thoracic surgeons and the anesthesiologist who performed the blocks had more than 5 years of dedicated clinical experience with their respective paravertebral block techniques, ensuring comparable operator expertise across groups.

### 2.5. Anesthesia Protocol and Surgical Procedure

All patients received standardized thoracic anesthesia in accordance with ERAS guidelines [1]. Anesthesia was induced with intravenous propofol and remifentanil, followed by rocuronium for intubation. A left-sided double-lumen tube was placed and verified using fiberoptic bronchoscopy. One-lung ventilation was conducted using a lung-protective strategy. Anesthesia was maintained with sevoflurane, a low-dose remifentanil infusion (0.05–0.15 µg/kg/min) titrated to hemodynamic targets, and rocuronium administration, in accordance with thoracic ERAS guidelines. Remifentanil was discontinued before wound closure to allow timely emergence. Hemodynamic changes were managed with standard vasoactive agents. All surgeries were performed using a four-port VATS technique by the same surgical team. A 3–5 cm utility incision was made at the fourth or fifth intercostal space, along with three additional thoracoscopic ports. Anesthetic agents were discontinued at the end of surgery, neuromuscular blockade was reversed with sugammadex, and extubation was performed following adequate recovery. Patients were then transferred to the PACU, and subsequently to the ward once the Aldrete score was ≥9.

### 2.6. Postoperative Analgesia Protocol

All patients received standardized multimodal analgesia. Preoperatively, the patients were educated on the use of the visual analog scale (VAS) and patient-controlled analgesia (PCA) [19]. All patients in both groups received the same standardized multimodal analgesic regimen, consisting of oral acetaminophen (600 mg) and celecoxib (200 mg) preoperatively; intravenous acetaminophen (1 g) and ketorolac (30 mg) at the end of surgery; and scheduled postoperative acetaminophen (600 mg every 6 h) and celecoxib (200 mg every 12 h). In the PACU, PCA was initiated with fentanyl 10 µg/kg in 100 mL saline without a basal infusion, delivering 2 mL boluses with a 10 min lockout. If VAS was ≥4 in the PACU, additional fentanyl (0.5–1 µg/kg) was administered. If pain persisted despite PCA, rescue fentanyl (0.5–1 µg/kg) was administered.

### 2.7. Outcomes

The primary outcome was dynamic VAS (VAS-D) during coughing at 1–6 h postoperatively, reflecting early mobilization-related pain. Secondary outcomes included resting VAS (VAS-R), VAS-D, and total fentanyl consumption at 0–1, 1–6, 6–24, and 24–48 h. VAS-R was assessed at rest and VAS-D during voluntary coughing. Patients were instructed preoperatively on the use of the 10 cm VAS, where 0 indicated no pain and 10 indicated the worst pain imaginable. VAS-R and VAS-D were assessed by a blinded observer at 1 h, 6 h, 24 h, and 48 h after surgery, with patients reporting the highest pain intensity experienced during each preceding interval (0–1 h, 1–6 h, 6–24 h, and 24–48 h). Fentanyl consumption—including PCA and rescue doses—were recorded at each interval. Block-related complications included total spinal anesthesia, neurological symptoms defined as sensory or motor deficits persisting beyond 24 h after surgery [20], vascular injury, and pneumothorax. Block success (dermatomal blockade) was evaluated after emergence from anesthesia in the PACU by the procedural team using pinprick and cold sensations. Dermatomal spread was mapped, and procedural time was recorded from needle insertion to completion of injection at T4 and T7.

### 2.8. Sample Size Calculation

The sample size was based on a non-inferiority design, with VAS-D at 1–6 h as the primary outcome. A minimal clinically important difference of 0.9 and SD of 1.3 (from institutional data) were assumed [16]. With α = 0.025 (one-sided) and 80% power, 33 patients per group were required. Considering a 10% dropout rate, 74 patients (37 per group) were enrolled.

### 2.9. Statistical Analysis

Non-inferiority of T-TPVB to U-TPVB was assessed based on VAS-D scores at 1–6 h, with non-inferiority defined as an upper 95% CI of the group difference was <0.9. Statistical analysis was performed using SPSS (version 24.0; IBM Corp., Armonk, NY, USA). Normality was assessed with the Kolmogorov–Smirnov test. Between-group comparisons used the *t*-test or Mann–Whitney U test for continuous variables, and the chi-square or Fisher’s exact tests for categorical variables. Changes in VAS and fentanyl over time were evaluated using repeated-measures analysis of variance (RM-ANOVA). All analyses primarily followed the intention-to-treat (ITT) principle, including all randomized patients, irrespective of block success or failure. In addition, a sensitivity analysis was performed excluding block failures to confirm the robustness of the non-inferiority findings. A one-sided α = 0.025 was used for non-inferiority; all other tests were two-sided (*p* < 0.05). Data are presented as mean ± SD or median [IQR], or *n* (%), as appropriate.

## 3. Results

### 3.1. Participant Flow

**Figure 1:**
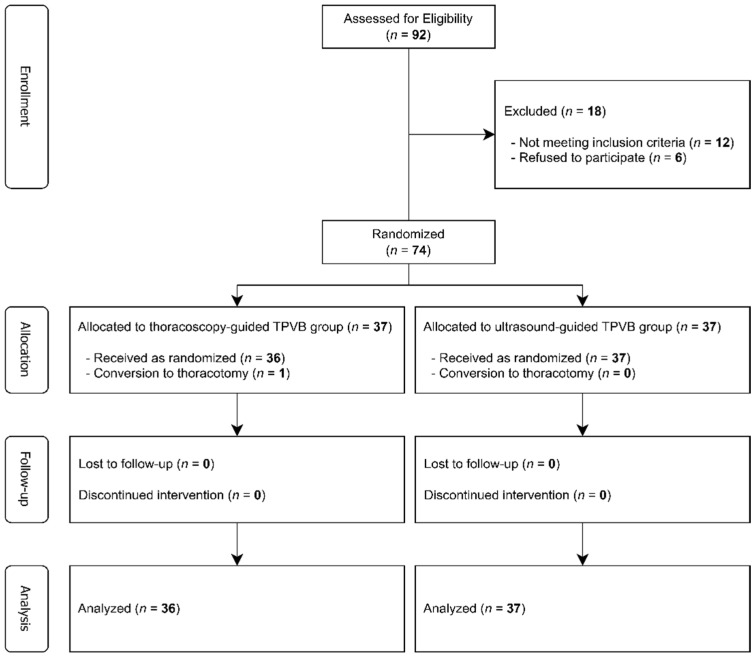
CONSORT flow diagram.

Of the 92 patients assessed for eligibility, 18 were excluded (12 did not meet inclusion criteria, 6 declined). The remaining 74 were randomly assigned to T-TPVB (*n* = 37) or U-TPVB (*n* = 37). In the T-TPVB group, 36 received the intervention, with one required conversion to thoracotomy. All 37 in the U-TPVB group receive the assigned intervention. Therefore, 36 and 37 patients in the T-TPVB and U-TPVB groups, respectively, were included in the ITT analysis (Figure 1).

### 3.2. Baseline Demographic and Clinical Characteristics

Baseline characteristics were well balanced between groups (Table 1). Mean age was 64.8 ± 8.4 years (T-TPVB) and 63.7 ± 7.9 years (U-TPVB). Distributions of sex, BMI, smoking history, hypertension, diabetes, and ASA classification were comparable. All absolute standardized differences were <0.25.

### 3.3. Surgical and Anesthetic Characteristics

Intraoperative details are presented in Table 2. Surgery duration was significantly shorter with T-TPVB than with U-TPVB (165.0 ± 11.2 min vs. 170.6 ± 10.6 min; *p* = 0.030), as was anesthesia duration (191.4 ± 9.5 min vs. 196.5 ± 10.9 min; *p* = 0.036). No significant differences were found in surgical laterality, procedure type, blood loss, utility port length, number or duration of chest tubes, or intraoperative remifentanil use.

### 3.4. Pain Outcomes

**Figure 2:**
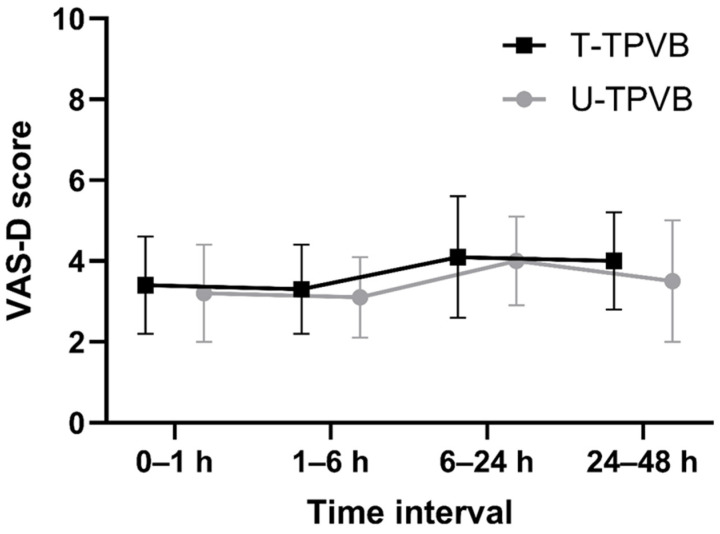
Dynamic visual analog scale scores over 48 h postoperatively. VAS-D, dynamic visual analog scale; T-TPVB, thoracoscopy-guided paravertebral block; U-TPVB, ultrasound-guided paravertebral block.

The primary outcome, VAS-D during coughing at 1–6 h postoperatively, 3.3 ± 1.1 (T-TPVB) and 3.1 ± 1.0 (U-TPVB; *p* = 0.342). The mean difference was 0.2 (95% CI: −0.3 to 0.7), within the predefined non-inferiority margin of 0.9. Based on the recently proposed Pd method quantifying the proportion of the confidence interval above the minimal clinically important difference [21], the Pd for the primary outcome was 0, indicating that none of the plausible treatment effects exceeded the predefined margin. Pain scores and opioid consumption over 48 h are presented in Table 3, with VAS-D trends in Figure 2.

RM-ANOVA showed a significant time effect on VAS-R, VAS-D, and fentanyl use (all *p* < 0.001), indicating variation over 48 h, peaking in the first 24 h. No significant group or group-by-time interaction effects were found (VAS-R, *p* = 0.473; VAS-D, *p* = 0.763; fentanyl use, *p* = 0.454), suggesting similar analgesic trajectories. Bonferroni-adjusted post hoc tests showed no significant differences at any time point (all corrected *p* > 0.0125), supporting the comparable analgesic efficacy and opioid-sparing effect of T-TPVB.

### 3.5. Block Procedure Characteristics and Complications

Block-related data are summarized in Table 4. Block failure was observed in two patients (5.7%) in the T-TPVB group and one patient (2.9%) in the U-TPVB group, indicating a low and comparable failure rate between groups. Procedural time was significantly shorter with T-TPVB (280.9 ± 23.4 s vs. 503.6 ± 26.4 s; *p* < 0.001). Median dermatomal spread was similar between groups (7 [IQR 6–7] vs. 7 [6,7,8]; *p* = 0.263). No cases of total spinal anesthesia or neurological complications were observed. Minor vascular injury occurred in two (5.6%) and one (2.7%) patients in the T-TPVB and U-TPVB group, respectively. One suspected pneumothorax, defined as air aspiration during needle placement under ultrasound guidance, was reported in the U-TPVB group (2.7%). Overall complication rates did not differ significantly.

### 3.6. Sensitivity Analysis

To test the robustness of the primary outcome, a sensitivity analysis excluded patients with block failure (two with T-TPVB and one with U-TPVB). Among the remaining patients, the mean VAS-D at 1–6 h was 3.3 ± 1.1 vs. 3.1 ± 1.0. The between-group mean difference was 0.2 (95% CI: −0.3 to 0.7), consistent with the predefined non-inferiority margin.

## 4. Discussion

In this single-center, randomized non-inferiority trial, surgeon-performed thoracoscopy-guided paravertebral block (T-TPVB) demonstrated analgesic efficacy comparable to that of ultrasound-guided TPVB (U-TPVB) in patients undergoing thoracoscopic anatomical lung resection. The primary outcome, dynamic pain during coughing at 1–6 h postoperatively, met the non-inferiority margin, and secondary outcomes including rest/dynamic pain trajectories and opioid consumption were also similar between groups. In addition, T-TPVB was associated with a significantly shorter procedural time, and complication rates were low and comparable. These findings suggest that T-TPVB may offer a practical and effective alternative for thoracic postoperative analgesia, with potential value in optimizing perioperative workflows. The study population was comparable to prior U-TPVB trials, supporting its use for non-inferiority analysis [17].

A growing body of literature has examined surgeon-performed T-TPVB as a practical alternative to conventional anesthesiologist-performed TPVB. Across randomized trials, cohort studies, and feasibility studies, T-TPVB has consistently demonstrated effective analgesia, high success rates, and low complication risk [11,12,13,14,15,22,23]. Consistent with this evidence, our findings reinforce the viability of T-TPVB as an alternative approach, supporting its integration into routine thoracic surgical practice.

Among prior studies, Chenesseau et al. demonstrated the non-inferiority of T-TPVB to U-TPVB in terms of 48 h opioid consumption [15], in patients undergoing minimally invasive thoracic surgery including both VATS and robotic approaches, while Xu et al. reported shorter procedural time and higher first-attempt success with T-TPVB, though their endpoints were primarily procedural [14]. It should be noted, however, that the technical approaches described in these prior studies were not identical: Chenesseau et al. used a percutaneous injection with thoracoscopic confirmation, whereas both Xu et al. and our trial performed a direct intrathoracic injection through the utility port. These differences reflect methodological variation across T-TPVB techniques but do not alter the shared goal of visually guided paravertebral deposition. These findings support the feasibility of T-TPVB but highlight the need for patient-centered outcome measures. Recent consensus guidelines recommend prioritizing patient-centered outcomes, particularly activity-related pain, at standardized time points [24,25]. Accordingly, our trial selected dynamic pain during coughing at 1–6 h as the primary endpoint, capturing a critical window for early mobilization and respiratory function. This design builds on previous studies while aligning with contemporary pain research priorities. Nevertheless, because the analgesic effect of single-shot paravertebral block is typically short-lived, pain intensity may increase beyond this early period; future approaches such as continuous infusion, long-acting formulations, or adjuvant-enhanced techniques could help extend analgesic duration beyond 6 h.

In addition to its analgesic efficacy, T-TPVB offers practical advantages that enhance clinical applicability. In our study, procedural time was significantly shorter, likely due to direct thoracoscopic visualization, which allows real-time anatomical identification without the need for ultrasound equipment or patient repositioning [26]. Performed through the utility port at the end of surgery, the block integrates seamlessly into the surgical workflow without prolonging anesthesia or turnover time. Unlike ultrasound-guided techniques, T-TPVB does not require specialized training in regional anesthesia, making it accessible to thoracic surgeons without prior experience in nerve blocks. The technique relies on clear thoracoscopic landmarks, enabling intuitive needle placement and visual confirmation of injectate spread [27]. As such, it may be feasible even in low-volume community hospitals or among less experienced surgeons, provided basic training in thoracoscopic anatomy and block principles. These logistical and educational advantages support T-TPVB as a scalable and practical alternative, particularly in settings with limited access to ultrasound, anesthesia personnel [28,29,30], or in patients with challenging anatomy (e.g., obesity, scoliosis, poor acoustic windows) [18].

These findings have important implications for perioperative care. With comparable efficacy and low complication rates, T-TPVB allows surgeons to contribute directly to multimodal pain management, particularly in settings with limited access to ultrasound or regional anesthesia services [28,29,30]. Incorporating T-TPVB into ERAS protocols may enhance the scalability of analgesic delivery without compromising quality [14,22]. As minimally invasive thoracic surgery becomes increasingly common, a surgeon-performed block that requires no extra equipment or personnel can streamline workflows and support value-based care [11,23].

In the present study, pain scores increased after 24 h, reflecting the limited duration of single-injection paravertebral block analgesia. Although both techniques provided comparable early pain relief, the VAS values at 24–48 h exceeded the threshold of <33 mm commonly regarded as acceptable for postoperative pain control [19]. This suggests that additional strategies such as continuous infusion, long-acting formulations, or adjuvant combinations may be necessary to extend analgesic coverage. Moreover, persistent moderate pain intensity (VAS > 40 mm) during the early postoperative period has been associated with increased risk of chronic postsurgical pain and psychological distress [31], underscoring the importance of optimizing multimodal analgesia beyond the initial block duration.

In accordance with current perioperative pain guidelines (e.g., PROSPECT and ERAS) [1,10], this study incorporated TPVB as a key component of multimodal analgesia for thoracic surgery. To balance efficacy and feasibility, we adopted a protocol of 10 mL of 0.5% ropivacaine at T4 and T7, administered at the end of surgery. This approach is supported by prior studies indicating that multi-level injections enhance segmental spread and sensory reliability [18,20], and that post-surgical block placement provides superior analgesia compared to intraoperative timing [32].

A key methodological strength of this study was the strict standardization of block parameters across both groups. Unlike prior trials with variations in timing, level, or volume [14,15], our protocol maintained identical settings for thoracic level, dose, concentration and timing. This minimized procedural variability and allowed isolation of the block technique’s effect, thereby enhancing the internal validity and interpretability of the non-inferiority findings.

This study had several limitations. First, although the sample size was adequately powered for the primary endpoint, it may have been underpowered to detect subtle differences in secondary outcomes or rare complications. Nevertheless, consistent trends across secondary measures support the robustness of our findings. Second, this was a single-center study conducted at a high-volume academic hospital, which may limit generalizability. However, the standardized protocol and pragmatic design enhance its relevance to real-world settings. Third, blinding of proceduralists was not feasible due to the nature of the intervention, introducing potential performance bias. To mitigate this, postoperative assessments were conducted by a blinded independent observer. Finally, pain scores were self-reported and inherently subjective. However, data were collected at predefined intervals using standardized instructions, minimizing variability. Objective nociceptive monitoring was not employed, as it remains investigational and lacks validation for routine clinical use [33].

## 5. Conclusions

In this randomized non-inferiority trial, surgeon-performed T-TPVB provided analgesia comparable to anesthesiologist-performed U-TPVB in patients undergoing VATS. Pain scores and opioid consumption were similar between groups, while procedural time was significantly shorter with T-TPVB. Both techniques were associated with low complication rates. These findings support T-TPVB as a safe and efficient option for postoperative analgesia in VATS. Its seamless integration into surgical workflow and reduced reliance on additional equipment or personnel may facilitate broader adoption within enhanced recovery protocols. Further multicenter trials are warranted to validate these findings and better define the role of surgeon-delivered regional blocks in enhanced recovery protocols.

## Figures and Tables

**Table 1:** Baseline demographic and clinical characteristics.

	T-TPVB (*n* = 36)	U-TPVB (*n* = 37)	ASD
Age, years	64.8 ± 8.4	63.7 ± 7.9	0.135
Body mass index, kg/m^2^	23.3 ± 2.2	23.7 ± 1.9	0.195
Male sex	25 (69.4%)	22 (59.5%)	0.209
Smoking history	16 (44.4%)	15 (40.5%)	
Hypertension	10 (27.8%)	13 (35.1%)	
Diabetes mellitus	7 (19.4%)	6 (16.2%)	0.079
ASA classification			0.159
I–II	23 (63.9%)	25 (67.6%)	
III	13 (36.1%)	12 (32.4%)	

Values are expressed as mean ± SD or *n* (%). ASA, American Society of Anesthesiologists; T-TPVB, thoracoscopy-guided paravertebral block; U-TPVB, ultrasound-guided paravertebral block.

**Table 2:** Intraoperative surgical and anesthetic characteristics.

	T-TPVB (*n* = 36)	U-TPVB (*n* = 37)	ASD
Surgery duration, min	165.0 ± 11.2	170.6 ± 10.6	0.514
Anesthesia duration, min	191.4 ± 9.5	196.5 ± 10.9	0.499
Laterality			0.135
Right	22 (61.1%)	25 (67.6%)	
Left	14 (38.9%)	12 (32.4%)	
Procedure			0.018
Lobectomy	25 (69.4%)	26 (70.3%)	
Segmentectomy	11 (30.6%)	11 (29.7%)	
Estimated blood loss, mL	125.6 ± 34.6	120.6 ± 34.0	0.146
Utility incision length, cm	4.3 ± 0.2	4.4 ± 0.3	0.392
Chest tube number			0.102
1	32 (88.9%)	34 (91.9%)	
≥2	4 (11.1%)	3 (8.1%)	
Chest tube duration			0.112
<24 h	28 (77.8%)	27 (73.0%)	
≥24 h	8 (22.2%)	10 (27.0%)	
Remifentanil consumption, µg/kg/min	0.10 ± 0.03	0.11 ± 0.04	0.283

Values are expressed as mean ± SD or *n* (%). T-TPVB, thoracoscopy-guided paravertebral block; U-TPVB, ultrasound-guided paravertebral block.

**Table 3:** Postoperative pain scores and fentanyl consumption.

		T-TPVB (*n* = 36)	U-TPVB (*n* = 37)	Mean Difference (95% CI)	*p*-Value
0–1 h	VAS-R	2.7 ± 1.3	2.4 ± 1.0	0.3 (−0.2 to 0.8)	0.233
	VAS-D	3.4 ± 1.2	3.2 ± 1.2	0.2 (−0.4 to 0.8)	0.514
	Fentanyl, µg	18.6 ± 5.9	16.9 ± 5.4	1.7 (−1.0 to 4.3)	0.213
1–6 h	VAS-R	2.9 ± 1.2	2.6 ± 0.9	0.3 (−0.2 to 0.7)	0.284
	VAS-D	3.3 ± 1.1	3.1 ± 1.0	0.2 (−0.3 to 0.7)	0.342
	Fentanyl, µg	17.9 ± 6.0	16.3 ± 5.8	1.5 (−1.2 to 4.3)	0.271
6–24 h	VAS-R	3.6 ± 1.2	3.7 ± 0.9	−0.0 (−0.5 to 0.5)	0.877
	VAS-D	4.1 ± 1.5	4.0 ± 1.1	0.1 (−0.5 to 0.7)	0.801
	Fentanyl, µg	27.6 ± 4.6	28.6 ± 6.9	−1.0 (−3.7 to 1.8)	0.484
24–48 h	VAS-R	2.7 ± 1.0	2.9 ± 1.0	−0.2 (−0.6 to 0.3)	0.502
	VAS-D	4.0 ± 1.2	3.5 ± 1.5	0.5 (−0.1 to 1.2)	0.124
	Fentanyl, µg	35.1 ± 5.9	34.3 ± 4.8	0.7 (−1.8 to 3.2)	0.554
	Cumulative fentanyl, µg	99.1 ± 11.5	96.1 ± 13.2	3.0 (−2.8 to 8.8)	0.310

Values are expressed as mean ± SD. T-TPVB, thoracoscopy-guided paravertebral block; U-TPVB, ultrasound-guided paravertebral block; VAS-R, resting visual analog scale; VAS-D, dynamic visual analog scale.

**Table 4:** Block procedure characteristics and related complications.

	T-TPVB (*n* = 36)	U-TPVB (*n* = 37)	*p*-Value
Block failure	2 (5.6%)	1 (2.7%)	0.981
Procedural time, s	280.9 ± 23.4	503.6 ± 26.4	<0.001
Dermatome spread, median [IQR]	7 [6, 7]	7 [6, 8]	0.263
Total spinal anesthesia	0 (0.0%)	0 (0.0%)	>0.99
Neurological complications	0 (0.0%)	0 (0.0%)	>0.99
Vascular injury	2 (5.6%)	1 (2.7%)	0.981
Pneumothorax	0 (0.0%)	1 (2.7%)	>0.99

Values are expressed as mean ± SD, median [IQR], or *n* (%). T-TPVB, thoracoscopy-guided paravertebral block; U-TPVB, ultrasound-guided paravertebral block.

## Data Availability

The data used to support the findings of this study are available from the corresponding author upon request.

## Supplementary Materials

The following supporting information can be downloaded at: https://www.mdpi.com/article/10.3390/jcm14238493/s1, Video S1: Thoracoscopy-guided thoracic paravertebral block, Video S2: Ultrasound-guided thoracic paravertebral block.
